# Community navigation intervention for social needs in patients with abnormal mammography: a randomized controlled trial study protocol

**DOI:** 10.1186/s12889-025-24372-x

**Published:** 2025-08-26

**Authors:** Kirstin Beck, Mackenzie Mitchell, Andrea S. Wallace, Phoebe E. Freer, Bob Wong, Deanna Kepka, Melissa H. Watt, Sierra McNall, Alex Kolomaya, Anthony Ariotti, Madilyn Mulford, Tracy Onega, Elissa M. Ozanne

**Affiliations:** 1 Population Health Sciences Department, University of Utah, Salt Lake City, USA; 2 College of Nursing, University of Utah, Salt Lake City, USA; 3 Department of Radiology and Imaging Sciences, University of Utah, Salt Lake City, USA; 4 College of Nursing, University of Utah, Salt Lake City, USA; 5 College of Nursing and Huntsman Cancer Institute, University of Utah, Salt Lake City, USA; 6 Internal Medicine University of Utah, Salt Lake City, USA; 7 Population Health Sciences Department, University of Utah, Salt Lake City, USA; 8 Huntsman Cancer Institute, University of Utah, Salt Lake City, USA

**Keywords:** Breast cancer, Mammography, Screening, Follow-up care, Abnormal mammogram, Social needs

## Abstract

**Background:**

Effective follow-up care plays a critical role in enhancing breast cancer outcomes, as diagnosing breast cancer in earlier stages can improve mortality and ease treatment burden. Individuals with unmet social needs face greater barriers in completing their follow-up care (e.g., additional imaging, biopsies, and surgery). They are two times less likely to complete follow-up appointments. When patients do not complete follow-up appointments, they are at higher risk of detecting breast cancer at a later stage. We aim to examine how a social needs intervention via community navigation may (1) address patients’ social needs, and (2) improve the completion of recommended medical care following an abnormal mammography.

**Methods:**

This is a single-center randomized controlled trial taking place in the Intermountain West. We plan to enroll 1,450 participants throughout the study. Women with abnormal mammography results will be invited to complete a validated, 10-item social needs screening assessment in English or Spanish. Eligible patients will be at least 18 years of age, speak English or Spanish, and indicate at least one social need. Participants will be randomized to passive or active social needs categories. Those in the active group will be called by 211 United Way services (211), a service which connects callers to community resources. The passive group will not receive a call from 211. Instead, they will be provided with information to contact 211 independently if they choose to. Primary outcomes are: (1) change in social needs, and (2) follow-up of abnormal mammography results, and will be assessed through follow-up surveys and chart reviews. Interviews and focus group discussions will explore how social needs influence patients’ adherence to recommended follow-up after an abnormal mammography result, and the role of community service navigation assistance in that follow-up.

**Discussion:**

This study utilizes a 10-item social needs assessment and community navigation services to address social needs that prevent women from receiving timely follow-up after an abnormal mammogram screening. Our goal is to improve breast cancer follow-up outcomes for women with social needs.

**Trial registration:**

The study was pre-registered at ClinicalTrials.gov on March 12, 2024, under identifier NCT06305312.

## Background

An estimated 42,680 people in the U.S. will die from breast cancer in 2025 [1]. Access to mammogram services, follow-up care, and early detection are associated with higher survival rates [2–6]. After an abnormal mammogram result, recommended follow-up care may include additional diagnostic imaging, a biopsy, or a visit with a healthcare provider. Receiving this follow-up care is crucial for improved breast cancer outcomes, as diagnosing breast cancer in earlier stages can improve mortality and also ease treatment burden [7]. Individuals with social needs face greater barriers in completing their follow-up care (e.g., additional imaging, biopsies, and surgery [8]) and are half as likely to complete follow-up appointments [9].

Barriers to healthcare access can include insufficient housing, lack of transportation, prohibitive cost of healthcare visits, lack of social support, and need for childcare [10]. These factors, if unmet, are known as “social needs” [11]. The need to implement programs to ease the burden of social needs or improve access to resources is well documented [12]. While the impact of social needs on health outcomes is widely recognized, further work is needed to improve access to social needs resources and to increase patients’ ability to attend follow-up appointments [13]. 

Prior research has shown successful implementation of a 10-item social needs assessment called SINCERE, as well as outreach from a community service navigator in an emergency department setting [14–16]. SINCERE stands for Screener for Intensifying Community Referrals for Health. The 10 questions chosen for this measure are centered around social needs, that if unmet, make it difficult for patients to complete healthcare visits. To expand on this, the present research aims to apply social needs screening using the SINCERE assessment, and community support services for those who receive abnormal mammography results and need to complete subsequent episodes of care. In the context of this study, “episode completion” means that the patient completes the next steps in care after receiving an abnormal mammogram result as suggested by their medical team. These episodes can mean completing additional imaging, receiving a biopsy, or attending another clinic visit. We will compare participants who receive the 211 social services navigation vs. participants who are only given the phone number for 211 social services. Ultimately, we aim to improve breast cancer screening episode completion and early detection of breast cancer.

### Hypotheses and aims

Central hypothesis: Community services navigation combined with social needs referrals will increase use of social services and improve breast cancer screening follow-up compared to the passive process of social service referrals alone.

Aim 1: Test whether the use of a community services navigation intervention in conjunction with social service referrals decreases social needs compared to the referrals alone (usual care).

Aim 1 Hypothesis: Patients who receive community services navigation will have fewer social needs over time when compared to patients who receive standard of care process of social service referrals alone.

Aim 2: Determine the impact of the community services navigation intervention (211 services) on breast cancer screening episode completion and preventive care utilization among women with self-reported social needs.

Aim 2 Hypothesis: Patients who receive the community services navigation intervention will complete their breast screening episode of care, e.g. additional imaging, follow-up visits, or biopsy and recommended follow-up and preventative services more than women who do not.

Aim 3: Qualitatively examine multilevel facilitators and barriers to successful engagement with community services and episode completion among underserved women with an abnormal breast cancer screen and self-reported social needs and explore the role of community service navigation.

## Methods and design

### Study design

**Fig. 1:**
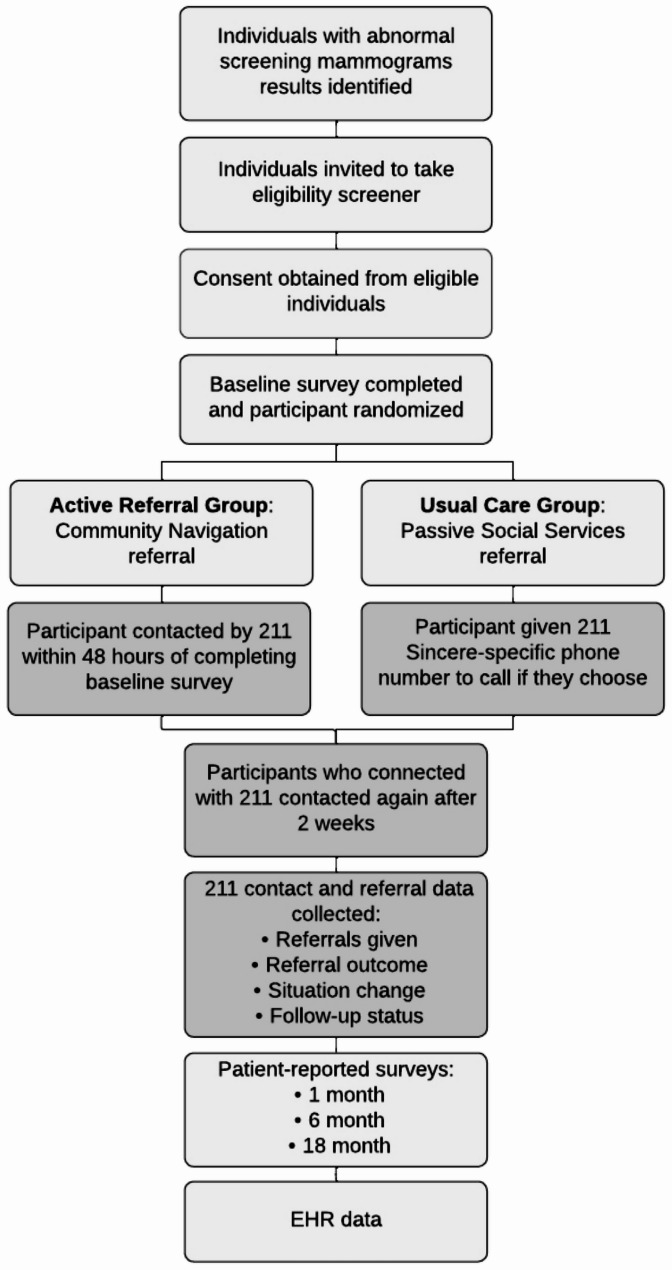
B-Sincere study schema

We will conduct a randomized controlled trial (RCT) within a mixed-methods sequential explanatory study [17]. We will use a single-center RCT to assess how community services navigation from United Way 211 Community Services (hereafter referred to as 211), impacts social needs and breast cancer screening episode completion. This will be followed by a qualitative phase designed to help explain the quantitative results obtained in the RCT. We will recruit 1,450 patients from multiple mammography clinics within the University of Utah health system, including mobile buses that serve rural Utah populations. The study protocol was reviewed and approved by the University of Utah IRB and is registered at clinicaltrials.gov NCT06305312. Figure 1 shows the study flow.

This clinical trial protocol follows the SPIRIT guidelines [18].

## Procedures

### Phase 1 – RCT

#### Recruitment and participation criteria

Eligible patients are those who are 18 and older, have completed a routine mammogram screening appointment and received an abnormal result (BI-RADS scores, e.g., scores 0, 3, 4, and 5), speak English or Spanish, and do not currently have a diagnosis of breast cancer. Individuals who have had a mammogram with an abnormal result will be recruited via study coordinator efforts as soon as their result is available in the medical record (usually within a few days of the exam). Participants will be invited to participate through multiple means (email, text, phone calls). Interested individuals will be asked to complete the 10-item SINCERE assessment to determine eligibility for the study. If patients indicate at least one social need on the SINCERE assessment, they will be invited to join the study. If they have no social needs, they are not deemed eligible for participation. Participants who indicate having a social need but decide they do not want to participate will be given 211’s contact information should they want to reach out independently. Any participant who requests to be withdrawn from the study can end their participation at any time by requesting to be withdrawn by contacting study staff.

#### Intervention

After providing informed consent and authorization, all participants will be randomized using the REDCap randomization function, with equal allocation to two arms: the usual care group or the active referral group.

The usual care group is provided the contact information for 211 if they choose to use it, but 211 does not reach out to these participants. 211 will receive the information of all participants, including those in the usual care group. If a usual care group participant reaches out to 211, they will note this for study records.

211 will reach out to all patients randomized to the active referral group within 48 h of receiving their information via REDCap. The 211 team will discuss the reported social needs with participants and match them with appropriate community resources that can help address their social needs. The 211 teams will also follow up again at 2 weeks with participants to ask if the participant has connected with services and offer additional support connecting to the social service referrals as needed.

#### Data collection

Data will be gathered through patient-reported outcome measures and extracting data from the patient’s health record. Participants will be asked to complete surveys at baseline, 6, 12, and 18 months after baseline. Surveys will be distributed through a REDCap survey link sent to participants at the appropriate time point via text or email. The Dillman total design principles for survey follow-up will be used in our survey approach [19].

#### Outcomes

The primary outcomes for this study are (1) change in participants’ social needs, and (2) episode completion. Social needs are determined by the SINCERE assessment, which the participant takes at baseline, 1 month, 6 months, and 18 months [15]. Episode completion in regard to follow-up care will be determined by patient-reported surveys and by accessing the patient’s electronic health record (EHR) to determine if they attended their follow-up care appointment(s) (e.g. additional imaging, biopsy, meeting with a specialist, or starting breast cancer treatment). As in our prior work within the national PROSPR I consortium (Onega, MPI) [20], we define a breast screening episode follow-up within a specified time interval based on Breast Imaging-Reporting and Data System (BI-RADS) category [21, 22]; specifically: BI-RADS 0,4,5 – episode completion within 3 months; BI-RADS 3 – episode completion within 9 months. The BI-RADS 3 classification is used infrequently (~ 2% of patients nationally, and < 1% at the University of Utah), and therefore, we anticipate we will capture > 99% of follow-up at our 6-month data collection point [21]. Data on both episode completion and time to completion will be collected.

The secondary outcomes are (1) social service utilization and (2) prevention and screening behaviors relating to breast, cervical, colorectal, and colon cancers. Health outcomes include trust in the healthcare system [25], cancer worry [26], and social support [27]. These will be assessed through patient reported outcomes at various timepoints throughout study enrollment.

Social service utilization data is gathered through patient reported measures as well as reports from 211 on participant utilization of social services. 211 records outcome at follow up, referral outcome, and if the patient’s situation has changed. Prevention and screening behaviors include next annual mammogram, cervical cancer screening, and colorectal cancer screening. We will ask women to self-report, and will also examine EHR records to capture guideline-concordant use of wellness visits.

Individual characteristics are derived both from the EHR and patient reported outcomes. Individual characteristics reported from the EHR include the Charlson Comorbidity Index [28], need factors (breast cancer risk, estimated with the Tyrer-Cuzick risk model [29]), environmental characteristics such as rural vs. urban residence based on ZIP code (categorized in the 4-tier Rural Urban Commuting Area (RUCA) schema) [30], area deprivation index (ADI) or patients’ census tract of residence per geocoded EHR-based address [31]. Individual characteristics reported by the patient include reported availability of services, predisposing factors (age, language, race and ethnicity, health literacy [32–34]), enabling factors including health care access, global health [23], and depression [24].

#### Analysis

For the primary outcomes of change in participant level of social need and episode completion, we will use mixed effects modeling, assuming missing at random, to determine if patients who receive community services navigation have greater reduction in social needs and if patients who receive the intervention complete their breast cancer screening episode of care and recommended follow up care. We will examine the interaction between time (conditional on baseline, 6, and 18 months) and intervention. We will analyze the time to follow-up completion using Cox proportional hazard modeling.

For the secondary outcome of community resource use, we will use a similar mixed-effects two-level model and utilize a log link function for a negative binomial regression for the count of social services used. We assume that the number of services will not be normally or dichotomously distributed. For the other secondary outcome of screening behavior, we will use the same mixed-effects two level model with log link function for a negative binomial regression.

Participant demographics will be compared between groups via t-Test and Fisher exact testing, Chi-squared testing, or Wilcoxon-Mann-Whitney test where appropriate.

To determine the sample size, power and sensitivity analyses were conducted with G-Power 3.1. We used a RMANOVA model with 3 total measures as a conservative approach for our mixed-effects model. We assume power = 0.95, alpha = 0.05, and two-tailed significance testing. Results indicate a small effect size (Cohen’s d = 0.09) can be detected. Therefore, recruitment of 1,450 patients with an attrition rate of 20%, will result in an analyzed sample of 1,160, which is sufficient power to detect a clinically meaningful effect. Unpublished pilot data obtained from participants who reported they had a social need the average amount of social needs was 3.06 with a standard deviation = 2.08. We will have 95% power to detect a reduction of social needs to 2.87.

### Phase 2 - Qualitative analysis

In order to understand the perspectives of health care providers, we will conduct focus group discussions (FGDs) with multidisciplinary providers who work with patients who receive abnormal mammogram results. These discussions will last for about 1.5 h, and participants will be compensated for their time. These interviews will be facilitated by a professional with the Community Collaboration & Engagement Team at the University of Utah. Additionally, these discussions will be viewed by an observer who will note factors like body language in a written report. During these focus groups, we will discuss barriers and facilitators of engagement, barriers in follow-up, community service navigation, and what support may be needed to improve the process.

Additionally, we will randomly select 40 individuals from the RCT for individual in-depth interviews (IDIs) to understand the perspectives of patients who participated in the intervention group. These 40 patients will include: 10 patients who received social services through 211 and completed mammography follow up, 10 individuals who received social services through 211 and did not complete mammography follow up, 10 who did not use utilize 211 and did complete mammography follow up, and 10 who did not utilize 211 *and* did not complete mammography follow up. To capture the unique needs of Spanish-speaking participants, at least half of those interviewed will be individuals who primarily speak Spanish, with certified interpreters available for the interviews as needed.

Audio tapes of the FGDs and IDIs will be transcribed, and then a thematic analysis framework will be implemented.

## Discussion

Previous research has found that women undergoing screening mammography face social and financial barriers such as income, insurance costs, and place of residence [10]. Targeted interventions may help those with social needs and abnormal mammography findings receive appropriate follow-up care by connecting patients with social services [13]. The current study tests the use of a low-cost community service intervention, United Way 211, among participants who report social needs and have received abnormal mammogram results.

Should this intervention help overcome barriers to the follow-up process, we can improve care for those with social needs. If this intervention benefits participants, we can implement a system in which clinicians and other healthcare staff can provide resources for their patients that do not place further burden on the healthcare team by collaborating with community partners. By addressing community barriers to healthcare, we meet patients where they are in their lived contexts. Through this holistic lens, we can support our patients who need it most.

## Data Availability

No datasets were generated or analysed during the current study.
